# Regular maintenance appointments after non‐surgical scaling and root planing support periodontal health in patients with or without dry mouth: A retrospective study

**DOI:** 10.1002/cre2.401

**Published:** 2021-01-20

**Authors:** Taylor V. Sparrow, Peter C. Fritz, Philip J. Sullivan, Wendy E. Ward

**Affiliations:** ^1^ Department of Kinesiology, Faculty of Applied Health Sciences Brock University St. Catharines Ontario Canada; ^2^ Periodontal Wellness & Implant Surgery Fonthill Ontario Canada; ^3^ Center for Bone and Muscle Health Brock University St. Catharines Ontario Canada

**Keywords:** dry mouth, periodontal maintenance, probing depth, salivary flow rate, scaling and root planing

## Abstract

**Objective:**

Non‐surgical scaling and root planing (SRP), as an initial form of periodontal treatment, followed by ongoing periodontal maintenance appointments is necessary to manage periodontal disease and prevent tooth loss. Saliva also has an essential role in oral health though the relationship between low salivary flow and periodontal outcomes has not been extensively investigated. This study determined if patients with dry mouth have similar clinical outcomes as patients without dry mouth when receiving regular periodontal maintenance after SRP.

**Materials and methods:**

This is a retrospective study that investigated clinical periodontal outcomes in patients with (*n* = 34) or without (*n* = 85) dry mouth who had undergone SRP 1 to 5 years prior and had routine periodontal maintenance. The presence of dry mouth was established based on a patient's unstimulated salivary flow rate.

**Results:**

Probing depth for both patients with or without dry mouth was similar between groups and maintained 1 to 5 years following initial SRP. Improved probing depth achieved post‐SRP was sustained regardless of dry mouth status.

**Conclusion:**

Patients with or without dry mouth did not exhibit different probing depths.

## 1 INTRODUCTION

The effectiveness of non‐surgical scaling and root planing (SRP) followed by periodontal maintenance in preventing periodontal disease, and ultimately tooth loss, has been documented in several longitudinal studies (Axelsson & Lindhe, 1981; Becker et al., 1979; L. Chambrone et al., 2010; L. A. Chambrone & Chambrone, 2006; Lindhe & Nyman, 1984; McGuire, 1991; Sparrow et al., 2020). At risk populations for periodontal disease include patients with cardiovascular disease, diabetes, or those who smoke (Bergström et al., 2000; Nguyen et al., 2015; Westfelt et al., 1996). Another at risk population may include patients with dry mouth though there is a paucity of information regarding the association between dry mouth induced by prescription medication and periodontal health.

Given that the intake of prescription medication, some with known side‐effects that include dry mouth, it is important to determine whether such individuals undergoing periodontal maintenance experience similar benefits as patients without dry mouth (Turner & Ship, 2007). A lack of saliva – which may result from use of prescription medication ‐ increases the adherence of plaque and bacteria, thus possibly contributing to an increased risk of periodontal disease (Gupta et al., 2006). Saliva plays an essential role in oral health and has other beneficial functions such as lubrication and protection of the oral cavity, removal and flushing of debris, mastication and digestion, as well as prevention of tooth erosion, abrasion and dental caries (Humphrey & Williamson, 2001). Additionally, dry mouth symptoms include mild oral discomfort, bad breath, cracked lips, sore throat, burning mouth, chelitis, dysgeusia, the inability to retain removable prostheses and can cause difficulty eating, which leads to higher consumption of poor nutrient foods such as soft breads, cookies, and soda (Guggenheimer & Moore, 2003; Meisel et al., 2012).

The main objective of this study was to determine if patients with or without dry mouth have the same clinical periodontal outcome (number of sites with probing depth ≥ 4 mm) when receiving regular periodontal maintenance at 1–5 years post‐SRP. We hypothesized that patients who underwent routine periodontal maintenance appointments would have no difference in periodontal outcomes at 1–5 years post‐SRP because of the frequent disruption of the biofilm and removal of plaque with regular periodontal maintenance, regardless of dry mouth status.

## 2 MATERIAL AND METHODS

### 2.1 Study population and design

**FIGURE 1:**
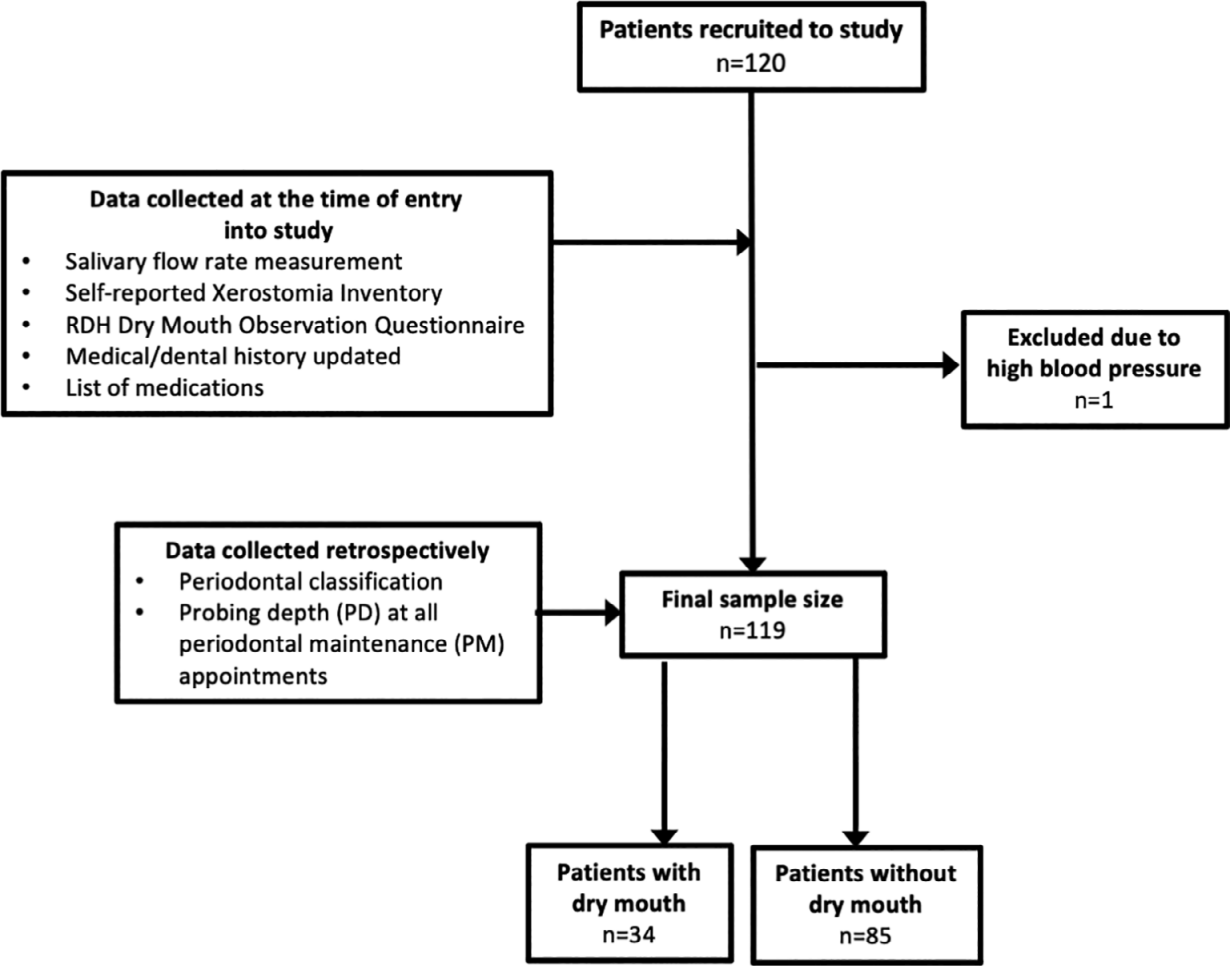
Flow diagram of study participants

This retrospective study took place at a periodontal specialty clinic in Fonthill, Ontario, Canada. Patients who had SRP within 1–5 years and who routinely visited the clinic for periodontal maintenance between July 2018 and October 2018 were invited to participate. One hundred twenty participants (convenience sample) were invited and 119 completed the study. One patient was referred for emergency treatment due to high blood pressure at the time of the periodontal maintenance appointment and thus clinical measures were not obtained (Figure 1). The presence or absence of dry mouth was measured at the “present‐day” periodontal maintenance appointment by measuring unstimulated salivary flow rate. Also, the self‐reported xerostomia inventory questionnaire and the Registered Dental Hygienist (RDH) dry mouth observation questionnaire were completed at this same time point (present‐day).

A patient with an unstimulated salivary flow rate less than 0.1 ml/min was categorized as having dry mouth. A patient with an unstimulated salivary flow rate of 0.1 ml/min or greater was considered to be without dry mouth. Patients were contacted 1 week prior to their scheduled periodontal maintenance appointment to confirm their appointment and invite them to the study. Patients who agreed were scheduled 15 min prior to their appointment. At the time of the appointment, patients provided written consent. The study protocol was approved by the human research ethics board at Brock University in St. Catharines, Ontario, Canada and registered with clinicaltrials.gov as NCT03649789.

### 2.2 Periodontal examination

Probing depth was measured at three time points, before SRP (pre‐SRP), 8–12 weeks following SRP (post‐SRP), and at the present‐day periodontal maintenance appointment. Data pertaining to dry mouth status, including salivary flow rate and information from the questionnaires, were collected at the present‐day appointment which took place between 1 and 5 years post‐SRP. Probing depth at pre‐SRP and post‐SRP were collected from the patient's dental charting history and probing depth at present‐day was collected at the time of saliva collection and completion of the questionnaires. Prior to measuring probing depth, the five hygienists were calibrated to use 25 N of pressure at six sites per tooth using a UNC15 Probe (Hu‐Friedy). These sites included mesiobuccal, buccal, distobuccal, mesiolingual, lingual, and distolingual. Average probing depths were calculated to compare among the three time points: pre‐SRP (1–5 years ago), post‐SRP (8 weeks following pre‐SRP) and present‐day.

### 2.3 Saliva collection

Patients provided a timed 5‐min unstimulated saliva sample to determine salivary flow rate. Patients were instructed to tilt their chin to their chest and allow for saliva to begin pooling at the front of their mouth. Following this, patients were instructed to guide their saliva through the Saliva Collection Aid, a straw‐like attachment apparatus, into the 2 ml cryovial. The volume (ml) of saliva collected per minute was used to calculate the salivary flow rate. Salimetrics protocol suggested a maximum of 5 min allocated, however, some patients were able to fill the cryovial before 5 min. Thus, the salivary flow rate was the amount of saliva in the cryovial divided by the minutes a patient used to provide the sample. A flow rate of 0.1 ml saliva/min or greater was considered adequate salivary flow rate while a flow rate of less than 0.1 ml saliva/min was considered inadequate and a patient was categorized as having dry mouth (Guggenheimer & Moore, 2003).

### 2.4 Self‐reported xerostomia inventory questionnaire

The Xerostomia Inventory was used as a self‐report questionnaire by the patient (Thomson et al., 1999; Thomson & Williams, 2000) to identify possible comparisons between salivary flow rate and self‐reported symptoms. At the beginning of their appointment, each patient completed this questionnaire prior to salivary flow rate collection. Each of the 11‐item statement was scored as follows: “Never” = 1 point, “Hardly Ever” = 2 points, “Occasionally” = 3 points, “Fairly Often” = 4 points, “Very Often” = 5 points. Scores of 11 equate to no symptoms of dry mouth reported, score of 12–33 were categorized to patients reporting mild to moderate dry mouth symptoms, and lastly scores greater than 33 equate to severe dry mouth symptoms.

### 2.5 Registered dental hygienist dry mouth observation questionnaire

Following a patient's periodontal maintenance appointment, the RDH completed the RDH dry mouth observation questionnaire (Plemons et al., 2014) that includes questions about the patient's salivation volume and consistency, observable symptoms dry mouth and use of dry mouth aids ‐ to assess the potential association between a patient's unstimulated salivary flow rate and dry mouth observations made by the RDH. A symptom score was calculated from the list of observable symptoms, with 1 observed symptom equating to 1 point for a possible total of 14 points.

### 2.6 Assessment of covariates

Patient height and weight were collected at the time of saliva collection to determine their body mass index (BMI; in kg/m^2^). Additionally, information about sex, age, and smoking status were obtained from the patient's updated medical history forms. These covariates were assessed as they have previously been found to be associated with periodontal disease. Specifically, a patient who has a higher BMI, is male and/or is a smoker, has a higher prevalence of periodontal disease (Al‐Shammari et al., 2005; Al‐Zahrani et al., 2003; Patel et al., 2012).

### 2.7 Statistical analysis

Probing depth and patient characteristics were analyzed by a repeated measures analysis of variance and independent *t*‐test to determine if there were significant differences for continuous variables such as age, number of medications consumed, probing depth and plaque index between patients with or without dry mouth. Chi‐square were performed to determine whether there is a significant difference between the expected and observed frequencies between patients with or without dry mouth for categorical variables such as sex, BMI, smoking status and type of medication. SPSS version 25 was used to analyze all statistical procedures (Armonk, New York). Statistical significance was defined as *p* < 0.05.

## 3 RESULTS

### 3.1 Patient characteristics

**FIGURE 2:**
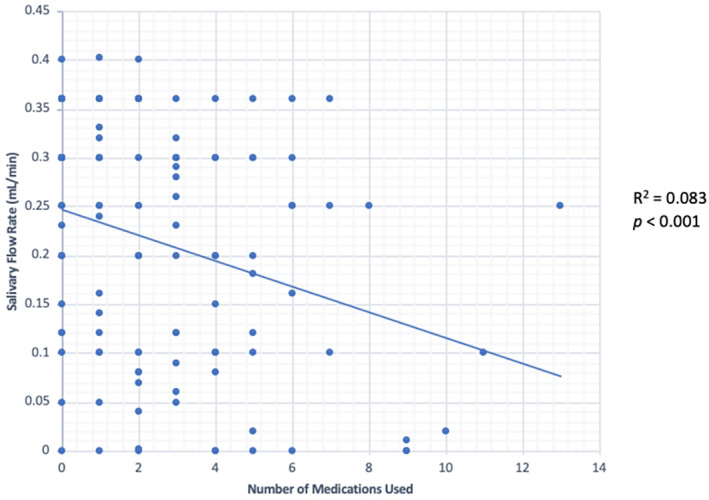
Correlation between the salivary flow rate and the number of medications used

Figure 1 illustrates patient recruitment. Of the 120 patients, 85 patients were determined to have adequate salivary flow rates and were considered to be without dry mouth. Thirty‐four patients were determined to have inadequate flow rate and were considered to be with dry mouth. One patient was excluded from present‐day appointment due to high blood pressure. This patient's saliva was collected, medical and dental history was updated, and consent was obtained. However, prior to periodontal maintenance their blood pressure was taken and exceeded the safe blood pressure recommendation for elective dental care. Therefore, the patient was dismissed and referred to a medical doctor. Table 1 shows the characteristics and clinical outcomes of patients with or without dry mouth. Age in both groups was similar. There was no significant difference between the number of males and females with dry mouth (*p* > 0.05). In regard to BMI, more patients without dry mouth had normal BMI compared to patients without dry mouth (*p* < 0.05). However, more patients with dry mouth were overweight (*p* < 0.001). There was no significant difference in the number of obese patients between groups. There were also no significant differences in smoking status (current smoker, former smoker, or never smoked) between groups (Table 1). Patients with dry mouth were using more medications than patients without dry mouth (*p* < 0.05). The number of medications used was also associated with a decreased salivary flow (*p* < 0.001) (Figure 2). When medications were categorized based on health condition (mental health, cardiovascular, diabetes, and osteoporosis) no significant differences were found (*p* > 0.05). As anticholinergic drugs are a significant predictor of dry mouth symptoms (Herbison et al., 2003), use of such drugs was assessed according to dry mouth status. No significant relationship was found between patients with or without dry mouth and use of anticholinergic drugs.

**TABLE 1:** Characteristics of patients with or without dry mouth^a^

Patient characteristics	Patients with dry mouth (*n* = 34)	Patients without dry mouth (*n* = 85)	*p* value^b^ ^,^ ^c^
Age, years	61.6 ± 8.7 (34–79)	61.6 ± 11.1 (33–90)	*p* > 0.05^b^
Sex, *n* (%)			
Males	13 (38.2)	43 (50)	*p* > 0.05^c^
Females	21 (61.8)	43 (50)	
BMI			
Normal	17 (50.0)	61 (70.9)	*p* < 0.05^c^
Overweight	15 (44.1)	20 (23.3)	*p* < 0.001^c^
Obese	2 (5.9)	5 (5.8)	*p* > 0.05^c^
All	23.28 ± 4.00 (14.05–38.85)	22.6 ± 4.23 (14.05–38.85)	*p* > 0.05^b^
Smoking status, *n* (%)			
Current smoker	9 (26.5)	18 (20.9)	*p* > 0.05^c^
Former smoker	12 (35.3)	34 (39.5)	*p* > 0.05^c^
Never smoked	13 (38.2)	34 (39.5)	*p* > 0.05^c^
Medication use, *n* (%)	31 (91.2)	58 (67.4)	*p* < *0*.05^c^
Mental Health	8 (23.5)	13 (15.1)	*p* > 0.05^c^
Cardiovascular	18 (52.9)	39 (45.3)	*p* > 0.05^c^
Diabetes	3 (8.8)	4 (4.7)	*p* > 0.05^c^
Osteoporosis	3 (8.8)	7 (8.1)	*p* > 0.05^c^
Number of Medications	3.8 ± 3.0 (0–11)	2.2 ± 2.4 (0–13)	*p* > 0.05^b^
Anticholinergic drugs,^d^ *n* (%)	9 (26.5)	13 (15.1)	*p* > 0.05^c^

^a^ Data are expressed as mean ± standard deviation, range as (lower and upper values) for continuous variables, and *n* (%) for categorical variables.

^b^ Independent samples *t*‐test was used to analyze group significance for continuous variables.

^c^ Chi‐squared test was used to analyze group significance for categorical variables.

^d^ Anticholinergic drugs treat a variety of conditions including chronic obstructive pulmonary disease, incontinence, gastrointestinal disorders, respiratory conditions, Parkinson's disease and mental health disorders.

### 3.2 Average probing depth for patients with or without dry mouth

There were no significant differences in average probing depth at each of the three time points between groups (*p* > 0.05) (Table 2). However, as expected, probing depth at pre‐SRP was significantly higher than probing depth at both post‐SRP and present‐day for both groups, regardless of dry mouth status (*p* < 0.05) (Table 2). There were no differences between groups (with dry mouth or without dry mouth) for number of sites ≥4 mm at pre‐SRP, post‐SRP or at the present‐day (*p* > 0.05). For plaque index, there was no difference between groups (*p* > 0.05). Given the new classification scheme introduced in 2018, periodontal diagnoses were categorized into stages and grades and examined for associations of dry mouth status. There were no differences between groups for Stage II Grade A, Stage II Grade B, Stage II Grade C, Stage III Grade B, Stage III Grade C, Stage IV Grade B, and Stage IV Grade C (*p* > 0.05) (Table 2). A significant difference was found among patients with a periodontal diagnosis of Stage III Grade A and their dry mouth status. More patients without dry mouth were classified as Stage III Grade A than patients with dry mouth (*p* < 0.05). In terms of current periodontal status, a higher number of patients without dry mouth had a current stable periodontal status than patients with dry mouth (*p* < 0.05). There were no differences (*p* > 0.05) between dry mouth groups in terms of patients with unstable periodontal disease or periodontal disease that was in remission. The number of patients that alternated periodontal maintenance appointments between the general dentist's clinic and the periodontal clinic did not significantly differ (*p* > 0.05) between patients with or without dry mouth (Table 2).

**TABLE 2:** Outcomes of periodontal health in patients with or without dry mouth^a^

Patient characteristics and clinical outcomes	Patients with dry mouth (*n* = 34)	Patients without dry mouth (*n* = 85)	*p* value^b^ ^,^ ^c^
Plaque Index^d^	40.1 ± 21.1 (10–100)	39.6 ± 19.6 (10–100)	*p* > 0.05^b^
Pre‐SRP PD, # of sites ≥ 4 mm	85.6 ± 34.7^e^ (13–144)	92.6 ± 40.6^e^ (9–183)	*p* > 0.05^b^
Post‐SRP PD, # of sites ≥ 4 mm	17.03 ± 11.8 (1–52)	14.3 ± 13.8 (0–72)	*p* > 0.05^b^
Present‐Day PD, # of sites ≥ 4 mm	16.5 ± 16.1 (0–69)	14.9 ± 13.7 (0–90)	*p* > 0.05^b^
Periodontal classification			
Stage II Grade A	3 (8.8)	2 (2.3)	*p* > 0.05^c^
Stage II Grade B	7 (20.6)	13 (15.1)	*p* > 0.05^c^
Stage II Grade C	0 (0)	1 (1.2)	*p* > 0.05^c^
Stage III Grade A	2 (5.9)	6 (7.0)	*p* < 0.05^c^
Stage III Grade B	13 (38.2)	39 (45.3)	*p* > 0.05^c^
Stage III Grade C	1 (2.9)	8 (9.3)	*p* > 0.05^c^
Stage IV Grade B	3 (8.8)	12 (14.0)	*p* > 0.05^c^
Stage IV Grade C	5 (14.7)	5 (5.8)	*p* > 0.05^c^
Current periodontal status			
In Remission	1 (2.9)	7 (8.1)	*p* > 0.05^c^
Stable	5 (14.7)	14 (16.3)	*p* < 0.05^c^
Unstable	28 (82.4)	65 (75.6)	*p* > 0.05^c^
Alternating Patient, *n* (%)	22 (64.7)	51 (59.3)	*p* > 0.05^c^
Non‐Alternating Patient, *n* (%)	12 (35.3)	35 (40.7)	

^a^ Data are expressed as mean ± standard deviation, range as (lower and upper values) for continuous variables, and *n* (%) for categorical variables.

^b^ Independent samples t‐test was used to analyze group significance for continuous variables.

^c^ Chi‐squared test was used to analyze group significance for categorical variables.

^d^ O'Leary Plaque Index.

^e^ Pre‐SRP PD was higher than post‐SRP and present day SRP regardless of dry mouth status.

### 3.3 Self‐reported xerostomia inventory questionnaire

There were no differences in dry mouth scores between patients with or without dry mouth (*p* > 0.05) (Table 3).

**TABLE 3:** Self‐reported score for dry mouth symptoms in patients with or without dry mouth^a^

Dry mouth score (0–55)		Patients with dry mouth (*n* = 34)^b^	Patients without dry mouth (*n* = 85)^b^	*p* value^c^
*n* (%)	0–11 (No dry mouth symptoms reported)	0 (0)	3 (3.5)	
	12–33 (Mild – Moderate Symptoms Reported)	29 (85.3)	79 (91.9)	
	> 33 (Severe symptoms reported)	5 (14.7)	4 (4.7)	
	Average score	26.97 ± 7.12 (14–44)	23.17 ± 7.34 (11–40)	*p* > 0.05^c^

^a^ Data are expressed as mean ± standard deviation, range as (lower and upper values) for continuous variables, and *n* (%) for categorical variables.

^b^ Dry Mouth status was determined through salivary flow rate samples collected at the patient's present day sanative therapy appointment (patients with a salivary flow rate of <0.1 ml/min samples were classified as having dry mouth).

^c^ Independent samples *t*‐test was used to analyze group significance.

### 3.4 RDH dry mouth observation questionnaire

There were significant differences in saliva volume, saliva consistency, and specific observable symptoms between patients with and without dry mouth (Table 4). The majority of patients without dry mouth were found to have a copious salivary volume and serous salivary consistency compared with patients with dry mouth (*p* < 0.001, *p* < 0.05). Patients with a dry mouth were more likely to have a slight or deficient salivary volume and a frothy salivary consistency than patients without dry mouth (*p* < 0.001, *p* < 0.05, *p* < 0.05). The number of patients with fissured tongue and tongue crenulations was higher among patients with dry mouth (*p* < 0.05, *p* < 0.05).

**TABLE 4:** Characteristics of patients with or without dry mouth using the registered dental hygienist dry mouth observation questionnaire^a^

Characteristics of patients	Patients with dry mouth (*n* = 34)^b^	Patients without dry mouth (*n* = 85)^b^	*p* value^c^ ^,^ ^d^
Saliva volume *n* (%)			
*Copious*	0 (0)	28 (32.6)	*p* < 0.001^c^
*Adequate*	19 (55.9)	50 (58.1)	*p* > *0.05* ^c^
*Slight*	12 (35.3)	8 (9.3)	*p* < 0.001^c^
*Deficient in Volume*	3 (8.8)	0 (0)	*p* < 0.05^c^
Saliva consistency *n* (%)			
*Serous*	14 (41.2)	60 (69.8)	*p* < 0.05^c^
*Sticky*	15 (44.1)	24 (27.9)	*p* > *0.05* ^c^
*Frothy*	5 (14.7)	2 (2.3)	*p* < 0.05^c^
Observable Symptoms			
*Dental Caries*	6 (17.6)	10 (11.6)	*p* > 0.05^c^
*Demineralization*	3 (8.8)	9 (10.5)	*p* > 0.05^c^
*Hypersensitivity*	8 (23.5)	27 (31.4)	*p* > 0.05^c^
*Mucositis*	1 (2.9)	1 (1.2)	*p* > 0.05^c^
*Candidiasis*	0 (0)	0 (0)	*p* > 0.05^c^
*Ulcerations*	1 (2.9)	3 (3.5)	*p >* 0.05^c^
*Non‐Specific Inflammation*	3 (8.8)	4 (4.7)	*p* > 0.05^c^
*Fissured Tongue*	9 (26.5)	7 (8.1)	*p* < 0.05^c^
*Crenulations*	8 (23.5)	8 (9.3)	*p* < 0.05^c^
*Dry Lips*	6 (17.6)	15 (17.4)	*p* > 0.05^c^
*Angular Chelitis*	3 (8.8)	2 (2.3)	*p* > 0.05^c^
*Halitosis*	3 (8.8)	5 (5.8)	*p >* 0.05^c^
*Food Retention*	18 (52.9)	30 (34.9)	*p* > 0.05^c^
*Loss of Papilla*	4 (11.8)	10 (11.6)	*p* > 0.05^c^
Symptom Score (*n*)^e^	2.32 ± 1.63 (0–6)	1.63 ± 1.54 (0–7)	*p* > 0.05^d^
Use of Dry Mouth Aids^f^	4 (11.8)	6 (7.0)	*p* > 0.05^c^

^a^ Data are expressed as mean ± standard deviation, range as (lower and upper values) for continuous variables, and *n* (%) for categorical variables.

^b^ Dry mouth status was determined through salivary flow rate samples collected at the patient's present day sanative therapy appointment (a flow rate of <0.1 ml/min was used to define if a patient had dry mouth).

^c^ Chi‐Square test was used to analyze group significance.

^d^ Independent samples *t*‐test was used to analyze group significance.

^e^ Symptoms included dental caries, enamel demineralization, tooth hypersensitivity, mucositis, oral candidiasis, traumatic gingival ulcerations, nonspecific gingival inflammation, fissured tongue, crenulations on tongue (scalloped borders), dry lips, angular chelitis, halitosis, food retention and debris on teeth and/or tongue, loss of papilla on tongue.

^f^ Examples of dry mouth aids utilized by patients include: Biotene Mouth Rinse, Biotene Toothpaste, Xyliment lozenges, sugarless gum, and frequent water consumption.

## 4 DISCUSSION

The study findings have supported the hypothesis that patients with dry mouth compared to patients without dry mouth who undergo routine periodontal maintenance appointments have no difference in periodontal outcomes at 1–5 years post‐SRP. Specifically, there were no significant differences in probing depth observed between patients with and without dry mouth at any of the three time points (pre‐SRP, post‐SRP, and present‐day). As expected, based on the fact that SRP is a proven procedure for managing periodontal disease, post‐SRP probing depth was markedly improved compared to pre‐SRP probing depth, and these benefits were maintained at up to 5 years post‐SRP, most likely due to routine periodontal maintenance appointments.

While we cannot determine if dry mouth was the result of medication use, on average, patients with dry mouth were more likely to be taking prescription medications than patients without dry mouth. It should be considered that as this study population engages in routine periodontal maintenance treatment, they may be generally healthier as they prioritize oral health care. This statement is supported by the fact that the Canadian Dental Association identified that almost 50% of lower‐income Canadians have a dental concern (decay, pain, or periodontal disease) compared to 26% of higher income Canadians (Canadian Dental Association, 2017). The adherence to periodontal maintenance treatment may also be preventing patients from needing additional medication as periodontal disease is related to various systemic disease (Canadian Dental Association, 2005). Approximately 30 percent of Canadians 65–79 years old use at least five prescription medications (Rotermann et al., 2014) and this level of medication use is higher than that of patient's in the present study in which the average number of medications used was an average of 3.8 and 2.2 for patients with or without dry mouth, respectively. One may argue that this is due to the overall better health among the study population.

When medication use was categorized into health conditions and specifically according to medications most commonly associated with dry mouth, no significant relationship was found. Perhaps a larger sample size is needed to evaluate the specific effects of individual drug types. Other possibilities that may have hindered a significant relationship may include the fact that we do not have information on the dosage and history of use – factors that influence the potential for side‐effects. As required by The College of Dental Hygienists of Ontario, medication use is always reviewed at the beginning of periodontal maintenance appointments though additional information regarding dose and how long the medication had been used were not included in this study due to the inaccuracy of self‐recall.

While the gold standard method in determining the presence of dry mouth is a salivary flow rate measurement (Navazesh & Kumar, 2008), we were also able to determine if there is an association between subjective questionnaires and objective salivary flow rates. When assessing a patient's self‐reported dry mouth symptoms through the Xerostomia Inventory, the average score did not differ by dry mouth status. Most patients, both with and without dry mouth, scored themselves between having mild to moderate dry mouth symptoms. Interestingly, approximately 5% of patients without dry mouth scored themselves as having severe dry mouth symptoms. Although the Xerostomia Inventory has shown validity in previous studies by correlating the questionnaire scores and oral observations, our study includes both salivary flow rates along with patient's self‐assessment of dry mouth symptoms using this questionnaire (Thomson et al., 1999; Thomson & Williams, 2000). The aim of including this questionnaire was to assess if there is a relationship between a well utilized dry mouth questionnaire and salivary flow rate. The findings did not strongly align. This may be due to the types of questions asked within the Xerostomia Inventory questionnaire. For example, the statement “the skin on my face feels dry” is one that may have various causes. Dry skin may arise from changes in climate, conditions such as eczema, frequency of showering, dehydration and even systemic conditions such as diabetes (Sekijima et al., 2018; Siddappa, 2003). Additionally, dry skin may be a result from aging, and as the mean age of the study population was 61 years of age, age may contribute to the reported dry skin (Siddappa, 2003). It was also noted that many patients required clarification as they completed the questionnaire; they reported challenges in completing the questionnaire due to unclear or confusing choices. The lack of agreement with the measurement of salivary flow rate highlight the importance of objectively measuring salivary flow rate to determine if a patient has dry mouth.

A questionnaire for RDH was also used to identify potential relationships between oral signs of dry mouth and salivary output. Significant differences were observed between patients with dry mouth and patient's without dry mouth in the amount of salivary volumes, defined as copious, slight and deficient. Moreover, no patients with dry mouth had a copious amount of saliva and no patients without dry mouth had a deficient saliva volume. Thus, this questionnaire was overall effective in determining the relationship between observed salivary volume and salivary output and may be a useful and efficient method to a tool to assess dry mouth status if it is not feasible to directly measure salivary flow rate. Based on this study population and the uncertainty and clarifications to complete the Xerostomia Inventory questionnaire, the RDH dry mouth observation questionnaire may be more appropriate and clinically suited in assessing symptoms of dry mouth and inadequate salivary production.

The findings of this study suggest that with routine periodontal maintenance, periodontal health can be maintained in patients who have undergone previous SRP, regardless of dry mouth status. No significant difference in probing depth was measured between the two groups and suggests that periodontal maintenance is an effective prevention method in patients with a dry mouth. Although no relationship was observed between the self‐reported questionnaire and dry mouth status, there is promise for detecting dry mouth status by a chair side questionnaire completed by a RDH. In conclusion, patients with or without dry mouth did not exhibit different probing depths.

## CONFLICT OF INTEREST

The authors declare that there are no conflicts of interest in this study.

## Data Availability

The data that support the findings of this study are available from the corresponding author upon reasonable request.
